# Aflatoxin M1 Determination in Whole Milk with Immersible Silicon Photonic Immunosensor

**DOI:** 10.3390/toxins17040165

**Published:** 2025-03-26

**Authors:** Dimitra Kourti, Michailia Angelopoulou, Eleni Makarona, Anastasios Economou, Panagiota Petrou, Konstantinos Misiakos, Sotirios Kakabakos

**Affiliations:** 1 Immunoassays–Immunosensors Lab, Institute of Nuclear & Radiological Sciences & Technology, Energy & Safety, NCSR “Demokritos”, GR-15341 Agia Paraskevi, Greece; d.kourti@rrp.demokritos.gr (D.K.); mikangel@ipta.demokritos.gr (M.A.); ypetrou@rrp.demokritos.gr (P.P.); 2 Analytical Chemistry Lab, Department of Chemistry, National and Kapodistrian University of Athens, GR-15771 Panepistimiopolis Zografou, Greece; aeconomo@chem.uoa.gr; 3 Institute of Nanoscience & Nanotechnology, NCSR “Demokritos”, GR-15341 Agia Paraskevi, Greece; e.makarona@inn.demokritos.gr (E.M.); k.misiakos@inn.demokritos.gr (K.M.)

**Keywords:** Mach–Zehnder interferometry, aflatoxin M1, immunosensor, whole milk, milk safety

## Abstract

Aflatoxin M1 (AFM1) appears in the milk of animals that have consumed feed contaminated with aflatoxin B1. AFM1 presence in milk is regulated by the European Commission, which has set the maximum allowable limits for adult and infant consumption to 50 and 25 pg/mL, respectively. Here, a rapid and sensitive method for detecting AFM1 in milk based on an immersible silicon photonic chip is presented. The chip features two U-shaped silicon nitride waveguides formed as Mach–Zehnder interferometers. One interferometer is functionalized with AFM1–bovine serum albumin conjugate and the other with BSA to serve as a blank. The chip is connected to a broad-band white LED and a spectrophotometer by a bifurcated optical fiber and an assay is performed by immersing the chip in a mixture of milk with the anti-AFM1 antibody. Then, the chip is sequentially immersed in biotinylated anti-rabbit IgG antibody and streptavidin solutions for signal enhancement. The assay is completed in 20 min and the detection limit for AFM1 in undiluted milk is 20 pg/mL. Given its analytical performance and the absence of pumps and fluidics that lead to a compact instrument design, the proposed immunosensor is ideal for the on-site detection of AFM1 in milk samples.

## 1. Introduction

**Figure 1:**
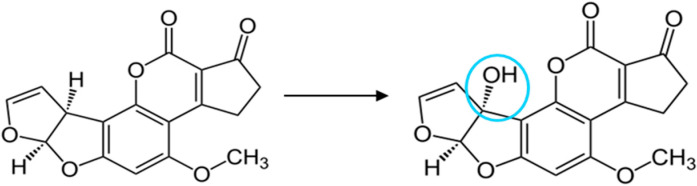
Chemical structures of AFB1 (**left**) and its metabolite AFM1 (**right**). The circle indicates the difference in the chemical structure of the two molecules.

Mycotoxins are produced by various types of fungi, which grow in soil, hay, decaying vegetation, and grains, especially under conditions of high humidity and temperature, and can contaminate crops, foodstuffs, and animal feed. One mycotoxin that raises high concern due to its mutagenic and carcinogenic function is aflatoxin B1 (AFB1). AFB1 is produced by certain species of Aspergillus fungi, like *Aspergillus flavus* and *Aspergillus parasiticus* [1]. When mammals consume feed contaminated with AFB1, it is metabolized in their liver by the action of hepatic microsomal cytochrome P450 monooxygenases (CYP) to aflatoxin M1 (AFM1) (Figure 1), which is then excreted in milk and can be transferred to humans through the consumption of contaminated dairy products [2]. Long-term exposure to AFM1 has been linked to several adverse health effects in humans, such as an increased risk of liver cancer, weakened immune function, and stunted growth in children. Due to these effects, AFM1 was first classified by the International Agency for Research on Cancer as a Group 2B carcinogen, i.e., as possibly carcinogenic to humans, and then reclassified as a Group 1 carcinogen based on further evidence about its effects on human health [3]. To ensure the protection of consumers from AFM1-contaminated dairy products, maximum allowable levels have been set by regulatory authorities. Hence, the European Union has set 25 and 50 pg/mL as the maximum allowable levels of AFM1 in milk for infants and adults, respectively [4]. These very strict limits, in combination with the obligation for dairy product producers to determine the AFM1 in milk upon delivery from farms and/or milk collection points and prior to the admission of milk into main facilities, have created the need for highly sensitive, accurate, and fast methods for the determination of AFM1 in milk samples.

To address the need for AFM1 detection in food and feed, various techniques have been developed, including chromatographic methods (High-Performance Liquid Chromatography [5] and Liquid Chromatography–Mass Spectrometry [6,7]) and immunochemical methods, such as Enzyme-Linked Immunosorbent Assay (ELISA) [8,9]. All these methods are highly effective for detecting AFM1 in various matrices with a high sensitivity and specificity, however, all these techniques are laboratory-bound and cannot be deployed for field testing. Amongst the established methods, only Lateral Flow Immunoassays (LFIAs) [10] are suitable for on-site determination, but they provide semi-quantitative results. Thus, there remains a need for cost-effective, portable, and accurate alternatives suitable for on-site testing.

Towards this direction, there has been an increasing interest in the development of biosensors for the detection of AFM1, mainly including electrochemical and optical transducers in combination with antibodies or aptamers as recognition molecules [11,12,13]. The majority of electrochemical sensors use aptamers as recognition molecules [14,15,16,17,18,19] for the binding of AFM1 onto the transducer surface and most of them do not require labels [14,15,16]. There are also reports of electrochemical AFM1 immunosensors [20,21] and cell-based sensors [22]. Although electrochemical sensors provide the detection sensitivity required to detect AFM1 in milk samples, most of them [15,16,17,18,21] require cumbersome sample pretreatment to avoid matrix effects. Optical sensors, on the other hand, are less prone to interference from the sample matrix, and, therefore, might be more suited for on-site determination [23]. The optical sensors developed so far for the determination of AFM1 include aptasensors based on different transduction principles, such as fluorimetry, Raman spectroscopy, colorimetry, and Localized Surface Plasmon Resonance (LSPR) [24,25,26,27,28]. Optical immunosensors based on Raman spectroscopy [29], SPR [30,31], White Light Reflectance Spectroscopy [32], or integrated onto silicon chip Mach–Zehnder interferometers [33,34] have been also reported. Additionally, an SPR-based sensor coated with a molecularly imprinted polymer nanofilm has also been reported in the literature [35]. Optical AFM1 sensors demonstrate, in their majority, detection limits lower than the maximum allowable concentration of AFM1 in milk. However, only a few can claim portability and ease of use suitable for on-site determination.

**Figure 2:**
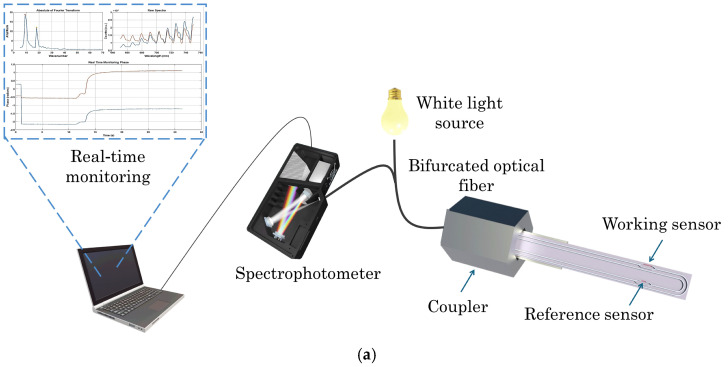

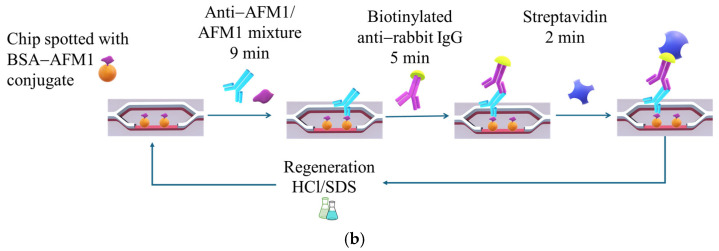
Schematic of (**a**) the optical set-up and the signal processing software and (**b**) the steps of AFM1 immunochemical determination with the immersible sensor.

In this work, an optical immunosensor for the detection of AFM1 in milk samples is developed. The immunosensor is based on silicon chips integrating two U-shaped silicon nitride waveguides formed as Mach–Zehnder interferometers (MZIs), with sensing window openings on one end of the chip and light input and output on the other. The chip is coupled to a white-light LED and a spectrometer for signal recording via a bifurcated fiber though a specially designed coupler (Figure 2a). The sensing window of one MZI is modified with an AFM1 conjugate with bovine serum albumin (working sensor) and the other with bovine serum albumin to compensate for potential matrix interferences (reference sensor). The assay is performed by sequentially immersing the chip in mixtures of calibrators or milk samples with an anti-AFM1-specific antibody followed by two signal enhancement steps, i.e., a reaction with a biotinylated secondary antibody and streptavidin (Figure 2b). All assay parameters are optimized, aiming for the highest possible detection sensitivity in the shortest assay time in order to facilitate the application of the sensor developed for the direct on-site determination of AFM1 in untreated milk samples. This work is in line with our previous works on the application of silicon chips integrating arrays of Mach–Zehnder interferometers for the determination of harmful substances in food samples, including AFM1 in dairy products [23,34]. A significant innovation of the proposed sensor is that there is no need for fluidics, external pumps, or liquid handling modules for the delivery of the solutions required to perform the assay. Instead of that, with the proposed sensor, the assay is performed by sequentially immersing the biofunctionalized chip tip in the assay solutions. By eliminating these components, the required instrumentation is reduced to a light source, a spectrometer, and a bifurcated optical fiber, all of which are integrated into a portable device for on-site measurements.

## 2. Results and Discussion

### 2.1. Matrix Effect

**Figure 3:**
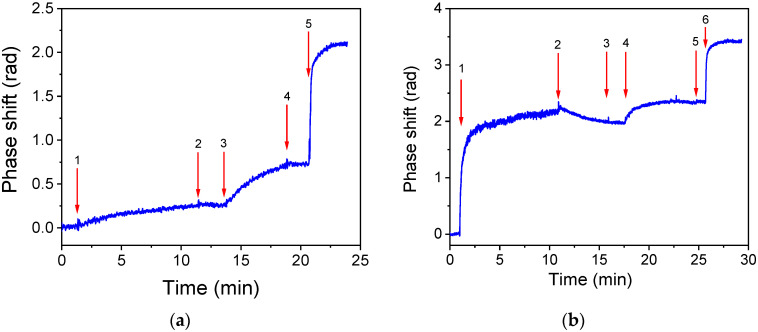
(**a**) Real-time net signal response for the zero AFM1 calibrator in assay buffer. The sequence of solutions is as follows: start to arrow 1: assay buffer; arrow 1 to 2: anti-AFM1 antibody solution/zero calibrator mixture in assay buffer; arrow 2 to 3: assay buffer; arrow 3 to 4: biotinylated secondary antibody in assay buffer; arrow 4 to 5: assay buffer; and arrow 5 to end: streptavidin in assay buffer. (**b**) Real-time net signal response for the zero AFM1 calibrator in undiluted cow milk. The sequence of solutions is as follows: start to arrow 1: assay buffer; arrow 1 to 2: anti-AFM1 antibody solution/zero calibrator mixture; arrow 2 to 3: washing buffer; arrow 3 to 4: assay buffer; arrow 4 to 5: biotinylated secondary antibody in assay buffer; arrow 5 to 6: assay buffer; and arrow 6 to end: streptavidin in assay buffer.

Optical transducers that monitor refractive index changes, such as MZIs, can be affected by complex matrices like milk. Additionally, the matrix may interfere with target analyte detection through non-specific interactions with the antibody or the biofunctionalized transducer surface. To assess the interference from the milk matrix on the photonic transducer used in this study, its responses using buffer or milk for the preparation of calibrators were compared. The chips used were spotted with a 100 μg/mL AFM1–BSA solution and the concentration of the antibody was 100 ng/mL. In Figure 3, the net chip response, i.e., the response of the working sensor corrected for the response of the reference sensor, is depicted for the assay buffer (Figure 3a) and milk (Figure 3b). The respective responses of the two sensors per chip, as well as the net chip responses, are provided in Figure S1. As shown in Figure 3a, when buffer was used, the primary immunoreaction, i.e., the reaction of the antibody with the immobilized antigen, generated a signal of approximately 0.26 rad (arrow 1 to 2). This signal increased to 0.72 rad (0.46 rad difference) after reacting with the biotinylated secondary antibody (arrow 3 to 4) and further increased to 2.09 rad (1.37 rad difference) after the streptavidin reaction (arrow 5 to the end).

In contrast, when milk was used in the primary immunoreaction, the signal reached 2.16 rad (arrow 1 to 2), indicating a significant contribution from the milk matrix that could not be fully eliminated by subtracting the reference sensor signal. The net signal dropped slightly after washing out the milk (arrow 2 to 3; Figure 3b), and increased by 0.38 rad after the reaction with the biotinylated secondary antibody and by 1.08 rad following the reaction with streptavidin. The differences in the responses observed for the different assay steps between the assay buffer and the milk indicate that components from the milk sample adsorbed onto the chip surface and were removed very slowly during washing. Using diluted samples, the matrix effect could be reduced, however, this would negatively influence the detection sensitivity. Therefore, the subsequent assay optimization study was performed using calibrators prepared in whole cow milk. Additionally, the signal received during the reaction with streptavidin was considered as the analytical signal of the assay to avoid matrix interferences.

### 2.2. Chemical Activation of the Chip

**Figure 4:**
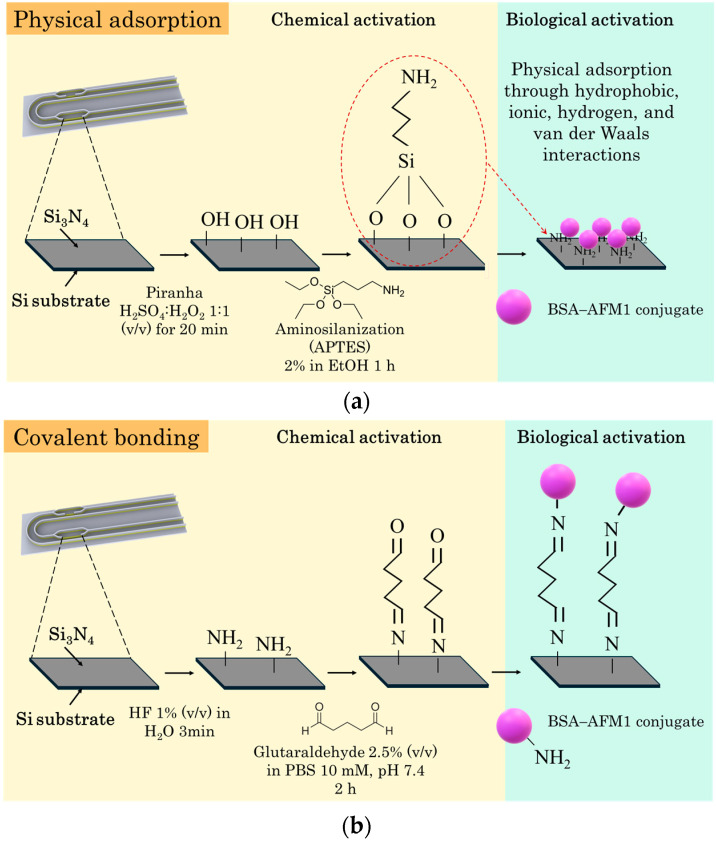
Schematic of the chemical functionalization of the working sensor with (**a**) APTES for BSA–AFM1 conjugate immobilization through physical adsorption and (**b**) HF/glutaraldehyde BSA–AFM1 conjugate immobilization through covalent bonding.

The chemical activation of the chip aimed to facilitate the attachment of BSA–AFM1 conjugate, preferably on the silicon nitride waveguide area exposed on the working sensor window. Two different chemical activation protocols and BSA–AFM1 immobilization procedures were compared. The first one involved the modification of the chips with 3-aminopropyl-triethoxy silane (APTES) after treatment with Piranha solution (1:1 *v/v* H_2_SO_4_/30% H_2_O_2_) to clean the surface and introduce silanol groups. The silanol groups reacted with APTES and, during the subsequent thermal curing, stable bonds were formed, as depicted in Figure 4a. Modification with APTES endowed the surface with amine groups which could be used for covalent bonding, but also made the surface suitable for the immobilization of proteins by physical adsorption, which provided higher sensor responses compared to covalent bonding [36]. The second approach consisted of treating the chips with HF solution, during which amine groups were created selectively on the Si_3_N_4_ [37], followed by the activation of the amine groups with glutaraldehyde to enable the immobilization of BSA–AFM1 conjugate by covalent bonding, as depicted in Figure 4b.

After chemical activation either with APTES or HF/glutaraldehyde, the working sensor was spotted with a 100 μg/mL AFM1–BSA conjugate solution, while the reference sensor was spotted with a 100 μg/mL BSA solution. For the assay, the chip was immersed in a mixture of zero calibrator with 100 ng/mL of anti-AFM1 antibody for 5 min, followed by immersion in a biotinylated anti-rabbit IgG solution for 5 min and a streptavidin solution for 2 min. It was found that the HF/glutaraldehyde activation of the chip provided approximately 15% higher signal values compared to chips modified with APTES. This finding is attributed to the fact that the HF/glutaraldehyde method led to the selective activation of the silicon nitride layer at the window areas of the two MZIs with respect to the surrounding silicon dioxide cladding layer, as opposed to chip modification with APTES. Therefore, the subsequent immunoreactions were also restricted on the sensing window area instead of on a larger area, thus leading to a signal increase. This is depicted in Figure S2a, where fluorescently labeled streptavidin was used to visualize the functionalized area under a fluorescence microscope in the case of activation with HF/glutaraldehyde (left) or APTES (right). Additionally, activation with HF/glutaraldehyde led to homogeneous coverage with the recognition biomolecule, i.e., the AFM1–BSA conjugate, of the silicon nitride area on the two MZI windows, as depicted in the image of Figure S2b. Therefore, treatment with HF/glutaraldehyde was selected for the chemical activation of the chips. The presence of amine groups on Si_3_N_4_ surfaces treated with HF was quantified following a previously published protocol [38]. In brief, the protocol included the reaction of surface amine groups with sulfo-SHPP [sulfosuccinimidyl-3-(4-hydroxypheynyl) propionate] to convert them into hydroxyphenyl moieties. Then, these moieties were quantified through the BCA protein assay method using an L-tyrosine calibration curve (Figure S3). For comparison reasons, the amine group contents of Si_3_N_4_ chips modified with APTES were also determined. The amine group content of HF-treated surfaces was determined at 2.23(±0.02) × 10^13^ groups/cm^2^, whereas the respective value for APTES treated surfaces was 1.22(±0.03) × 10^13^ groups/cm^2^, indicating that the HF treatment led to a higher concentration of amine groups per surface area compared to the APTES modification.

### 2.3. Assay Parameter Optimization

**Figure 5:**
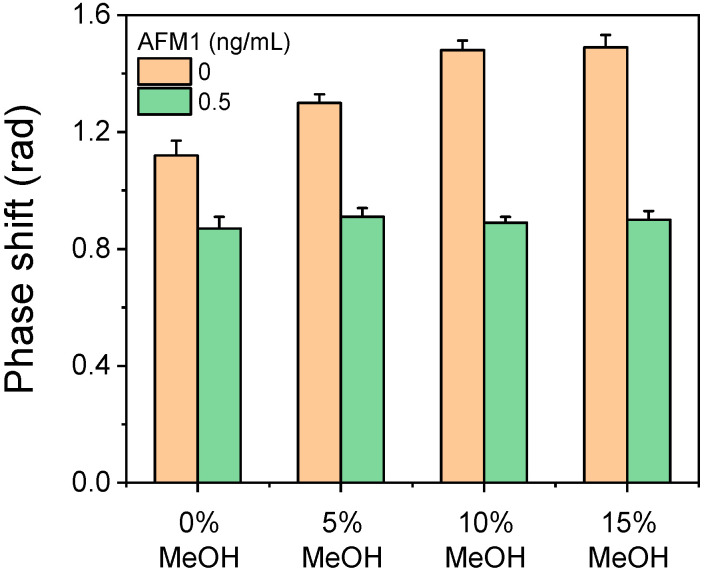
Net signals obtained from chips spotted with a 100 μg/mL BSA–AFM1 conjugate solution for the zero calibrator (orange columns) and a calibrator containing 0.5 ng/mL AFM1 (green columns) with respect to percent volume content of methanol in antibody dilution buffer. The anti-AFM1 antibody concentration used 100 ng/mL and the primary immunoreaction duration was 5 min. Each column corresponds to the mean of 3 chips ± SD.

The antibody dilution buffer composition was one of the critical assay parameters optimized with respect to the zero calibrator signal and the assay sensitivity. Two buffers were tested, 50 mM Tris-HCl, pH 7.8, and 50 mM phosphate-buffered saline (PBS), pH 7.4, both containing 0.9% (*w/v*) NaCl and 0.5% (*w/v*) BSA. The Tris-HCl buffer provided analytical signal values approximately 20% higher than those received for PBS and was, thus, selected for further experiments. Given the low water solubility of AFM1, the impact of adding methanol to the antibody dilution buffer on the zero calibrator signal was investigated. As shown in Figure 5, the addition of 10% (*v/v*) methanol to the antibody dilution buffer had two positive effects. Firstly, the zero calibrator signal increased by approximately 27% compared to that obtained with buffer without methanol. Furthermore, the assay sensitivity was improved, as indicated by the increase in the percentage of signal inhibition obtained for a calibrator containing 0.5 ng/mL of AFM1 from 24.5% in the absence of methanol to 40% in the presence of 10% *v/v* methanol in the antibody dilution buffer. The increased signal can be attributed to the fact that methanol helped to expose the AFM1 moieties immobilized on the sensor surface, as a protein conjugate, making them more accessible for binding to the antibody. Similarly, the enhanced detection sensitivity in the presence of methanol can be ascribed to the in situ extraction of AFM1 molecules from milk proteins, particularly caseins, that increased the concentration of available AFM1 molecules, thus facilitating competition with the AFM1 moieties of the immobilized conjugate for the binding sites of the antibody [39].

Based on these findings, we also tested the inclusion of 5% methanol in the buffer used for washing after the primary immunoreaction. It was found that 2 min of washing with this solution reduced the signal received during the primary immunoreaction due to the milk effect by approximately 50%, whereas washing without methanol for 5 min decreased the signal only by 8% (Figure 3b, arrow 2 to 3).

**Figure 6:**
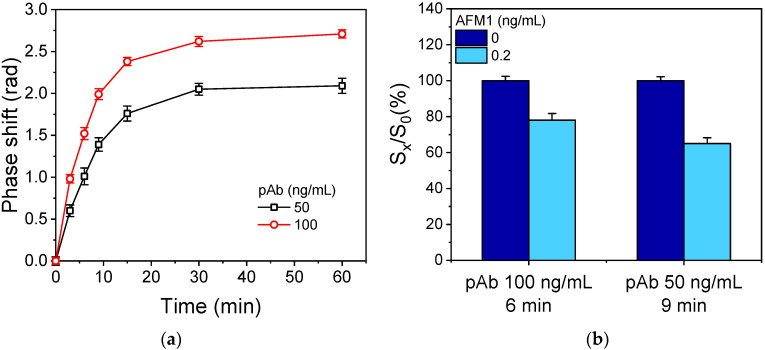
(**a**) Net zero calibrator signals obtained from chips spotted with a 50 μg/mL BSA–AFM1 conjugate and assayed with a 1:1 volume mixture of milk with anti-AFM1 antibody solution with concentration of 50 (black squares) or 100 ng/mL (red circles) for different assay times. (**b**) Percent signal values obtained for a calibrator containing 0.2 ng/mL AFM1 (light blue columns) with respect to zero calibrator (dark blue columns) employing two different combinations of the anti-AFM1 antibody concentration and primary immunoassay duration. Each value corresponds to the mean of 3 chips ± SD.

After the selection of the antibody dilution buffer, the concentration of the anti-AFM1 antibody and the primary immunoreaction time were optimized while keeping the concentrations and reaction times constant for the signal amplification steps (i.e., the secondary antibody and streptavidin reactions). As shown in Figure 6a, using either a 50 or 100 ng/mL anti-AFM1 antibody solution, the maximum plateau signal values were obtained for a primary immunoreaction time equal to or higher than 30 min. However, an adequate zero calibrator signal (approximately 1.5 rad) was achieved with either a 6 min reaction using 100 ng/mL or a 9 min reaction using 50 ng/mL of anti-AFM1 antibody. For the AFM1–BSA conjugate used for coating, the maximum zero calibrator signal plateau was reached with a concentration of 50 μg/mL (Figure S4). Thus, using a 50 μg/mL AFM1–BSA conjugate solution for coating, the sensitivity provided by a 6 min reaction using 100 ng/mL of anti-AFM1 antibody or a 9 min reaction using 50 ng/mL anti-AFM1 antibody was tested. As shown in Figure 6b, a 9 min primary immunoreaction duration using a 50 ng/mL anti-AFM1 antibody solution provided 35% inhibition for a calibrator containing 0.2 ng/mL of AFM1, while 22% inhibition was obtained for the same calibrator with a 6 min primary immunoreaction duration using a 100 ng/mL anti-AFM1 antibody solution. Thus, in the final protocol, a 50 ng/mL AFM1 antibody solution and a 9 min primary immunoreaction duration were adopted.

### 2.4. Analytical Characteristics

**Figure 7:**
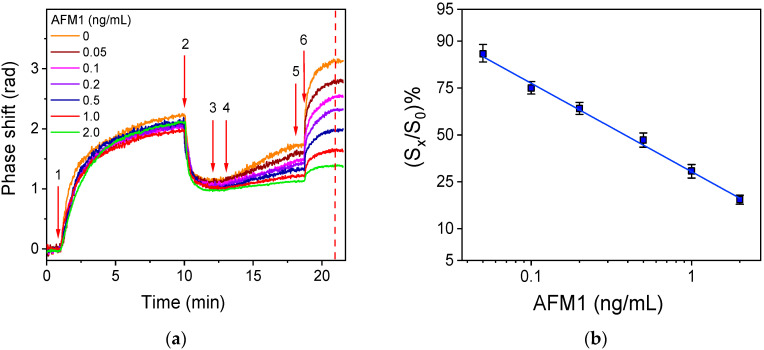
(**a**) Real-time signal responses of calibrators containing 0–2 ng/mL AFM1 in undiluted milk. The sequence of solutions are as follows: start to arrow 1: assay buffer/MeOH; arrow 1 to 2: anti-AFM1 antibody solution/zero calibrator mixture; arrow 2 to 3: washing buffer/MeOH; arrow 3 to 4: assay buffer; arrow 4 to 5: biotinylated secondary antibody in assay buffer; arrow 5 to 6: assay buffer; and arrow 6 to end: streptavidin in assay buffer. The analytical signal is calculated as the difference of response between the vertical red line (indicates the time point of 2 min reaction with streptavidin) and the response at arrow 6 (indicating the start of reaction with streptavidin). (**b**) Typical AFM1 calibration curves obtained with calibrators prepared in undiluted milk. Each point is the mean value of 3 measurements ± SD.

In Figure 7a, the net signals corresponding to different AFM1 calibrators prepared in cow milk are presented. Using the signals corresponding to the reaction with streptavidin, the calibration curve presented in Figure 7b was obtained. Additionally, the calibration curves obtained from calibrators prepared in sheep and goat milk were similar to those obtained from cow milk, demonstrating the potential of the method for AFM1 detection in milk from different animal species (Figure S5).

The detection limit (LOD) of the assay was calculated as the concentration corresponding to the percent signal equal to 100-3SD of the mean zero calibrator signal of 10 measurements and was found to be 20 pg/mL AFM1, with a linear dynamic range from 50 to 2000 pg/mL. This LOD is lower than the maximum allowable limits set by the EU for milk intended for both adult and infant consumption. The reproducibility of the method was evaluated through measurements of three fresh cow milk control samples prepared by spiking known amounts of AFM1 that covered the whole range of the calibration curve. The intra-assay coefficient of variation (CV) was determined by triplicate measurements of these samples on the same day, whereas the inter-assay CV was determined by duplicate measurements on 4 different days over a period of 1 month. The assay was reproducible with intra- and inter-assay CVs lower than 7.3 and 9.1%, respectively. Similar experiments were performed for control samples from fresh sheep and goat milk. The intra-assay and the inter-assay CVs were lower than 8.2 and 9.6% for the sheep and 7.7 and 9.3% for the goat milk, respectively. Additionally, the chip-to-chip variation was determined by assaying five chips from the same batch in a single day with respect to the zero calibrator signal. A coefficient of variation of less than 7.5% was determined. Moreover, the batch-to-batch variation was determined by assaying chips (in triplicate) from six different batches prepared over a period of 2 months. The coefficient of variation of the mean zero calibrator value determined for the different batches was up to 10.8%.

The accuracy of the assay was determined by recovery experiments with samples of fresh cow, sheep, and goat milk spiked with AFM1 at three different concentration levels (0.15, 0.4, and 1.5 ng/mL). The %recovery was calculated according to the following equation:

%Recovery = (AFM1 amount determined)/(AFM1 amount added) × 100%

It was found that the assay is accurate, with %recovery values ranging from 86.7 to 112% (Table 1).

The specificity of the assay was evaluated through cross-reactivity studies. In Figure S6, the AFM1 calibration curve along with inhibition curves obtained with calibrators of substances with a similar chemical structure to AFM1 are presented. The cross-reactivity values determined were 1.4% for aflatoxin B1 (AFB1), 0.30% for aflatoxin B2 (AFB2), and 0.12% for aflatoxin G1 (AFG1), indicating the high specificity of the proposed immunosensor against the targeted analyte.

### 2.5. Regeneration and Stability of the Sensor

**Figure 8:**
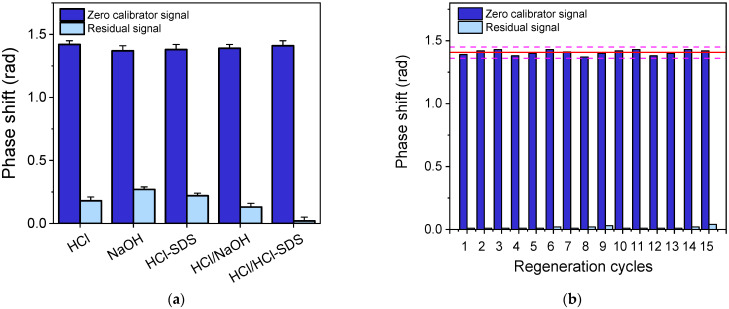
(**a**) Net zero calibrator signals obtained (blue columns) and the respective residual signals (light blue columns) obtained after treatment with different regeneration solutions. (**b**) Net zero calibrator signals obtained from a single chip after repetitive assay/regeneration cycles. The solid red corresponds to the mean value of the 15 measurements and dashed pink lines to mean value ± SD.

Photonic immunosensor chips can be reused after the completion of an assay cycle by disrupting the immunocomplexes formed and making the immobilized antigen available for a new assay cycle. To achieve this, various solutions, including 50 mM HCl, 40 mM NaOH, and a 0.5% (*w/v*) SDS solution, adjusted at pH 1.3 with 0.1 M HCl (HCl-SDS), as well as combination of HCI with either a NaOH or HCl-SDS solution, were investigated as regeneration buffers. In all cases, the chip was immersed in the regeneration solution for 2 min; when two regeneration solutions were used, a 1 min washing step was introduced between them. To select the optimum regeneration solution, it was necessary to determine the amount of anti-AFM1 antibody that remained on the chip surface after regeneration. For that reason, the residual signal was determined, i.e., the signal received after regeneration, by immersing the chip in a biotinylated anti-rabbit antibody and streptavidin solution. It was found that the use of two-step regeneration with a HCl and SDS-HCl solution almost completely removed the bound anti-AFM1 antibody molecules, as the residual signal was negligible, while, in all other cases, there was a substantial residual signal that would affect the next measurement (Figure 8a). The stability of the signal after multiple measurements/regeneration cycles with HCl/SDS-HCl was also evaluated. As shown in Figure 8b, the chips could be regenerated up to 15 times without any obvious effect on the analytical signal. Further assay/regeneration cycles led to an increased residual signal, which could affect the accuracy of the measurements.

Additionally, the stability of the biofunctionalized chips, which were stored at room temperature (RT), was assessed. The results showed that the biofunctionalized chips could be used for up to 3 months after spotting without any signal loss (Figure S7). In fact, the coefficient of variation of the mean values received from chips assayed in triplicate at different intervals within the 3-month period was 1.51%. Although a more elaborated investigation of sensor performance stability under different storage conditions might be necessary [40], the presented data are very promising, taking into account that no special treatment of the sensor was performed after the biomolecule spotting to help preserve their functionality.

### 2.6. Comparison with Label-Free Biosensors Reported in the Literature for AFM1 Detection in Dairy Products 

A comparison of the developed label-free MZI immunosensor with other label-free electrochemical or optical biosensors reported in the literature for the determination of AFM1 is provided in Table 2. An electrochemical sensor based on interdigitated Pt electrodes (IDEs) modified with an electropolymerized Fe_3_O_4_/polyaniline layer was functionalized after activation with glutaraldehyde with an aptamer specific for AFM1 [14]. The sensor was applied for direct AFM1 detection in buffer through cyclic and square wave voltammetry, achieving an LOD of 1.98 pg/mL after a 1 h reaction [14]. In another study, two different approaches for the electrochemical detection of AFM1 were investigated [15]. The first method involved gold electrodes modified with a polyamidoamine dendrimer layer to covalently bind an amine-terminated aptamer. The second involved the modification of the electrodes with neutravidin to enable the binding of a biotinylated aptamer [15]. Both approaches yielded similar results in terms of LOD, which was 8.47 pg/L, and AFM1 recovery in spiked milk samples, which was higher than 78%. Another report involved the electropolymerization of the neutral red dye onto glassy carbon electrodes (GCEs) in the presence of a polycarboxylated pillar[5]arene derivative to introduce carboxyl groups onto the electrode for the covalent binding of an aptamer for AFM1 [16]. The sensor was applied for the detection of AFM1 in cow and sheep milk and kefir after their dilution with methanol, achieving an LOD of 40 pg/mL. In another report, screen-printed carbon electrodes were modified with a nanocomposite of molybdenum disulfide (MoS_2_) quantum dots (QDs) and a zirconium-based metal–organic framework (UiO-66-NH_2_), followed by the covalent immobilization of an AFM1-specific antibody, and used to directly detect AFM1 with an LOD of 60 pg/mL following a 10 min reaction [20]. Furthermore, an impedimetric immunosensor was developed using silver (Ag) wire electrodes modified with 11-mercaptoundecanoic acid and a covalently bound antibody to detect AFM1 in milk samples, with an LOD of 1 pg/mL [21].

Regarding the label-free detection of AFM1 with optical sensors, a fiber optic localized SPR aptasensor modified with gold nanoparticle multimers to enhance the signal due to a hot spot effect created on the nanogaps between nanoparticles has been reported [27]. This sensor could detect AFM1 with an LOD of 50 pg/mL. Another sensor that combined LSPR and total internal reflection ellipsometry (TIRE) enabled the direct detection of AFM1 in buffer, with an LOD of 10 pg/mL [28]. SPR was also employed for AFM1 detection in milk and milk powder with an LOD of 100 pg/mL in 10 min, after the defatting and immunoaffinity isolation of AFM1 [30]. Furthermore, an interferometric immunosensor based on White Light Reflectance Spectroscopy, which monitored the changes in the biomolecular thickness on the transducer surface during reactions, was used to detect AFM1 through a competitive immunoassay, with an LOD of 6 pg/mL [32]. Another interferometric immunosensor, based on integration onto silicon asymmetric Mach–Zehnder interferometers (MZIs) functionalized with the Fab’ fragment of an anti-AFM1 antibody, was used for AFM1 detection in milk within 1.5 min with an LOD of 16.8 pg/mL, after defatting, 20 times pre-concentration, and column purification of the milk samples [33]. Moreover, silicon chips with arrays of ten silicon nitride waveguide MZIs integrated on the same chip along with respective broad-band light sources were used for the detection of AFM1 in plain and chocolate cow milk and yogurt following a competitive immunoassay format [34]. The LODs achieved were 5 pg/mL in plain and chocolate cow milk and 10 pg/mL in yogurt, and the assay was completed, in all cases, in 15 min [34].

Compared to the electrochemical and optical label-free apta- and immunosensors reported in the literature, the proposed immunosensor may not be the most sensitive, but the LOD achieved is lower than the maximum allowable limits of AFM1 in milk for infant and adult consumption. Additionally, one major advantage of the proposed immunosensor is that it does not require any milk sample pretreatment and can be applied to detect AFM1 in milk from different species. Moreover, compared with previous chips developed by our team and used to determine AFM1 in milk and dairy products, the proposed immunosensor has the following advantages. (a) Its immersible form eliminates the need for microfluidics and pumps, allowing for the construction of a small-size portable instrument for on-site determination. The lack of flow, however, might be the reason that the proposed immunosensor has a four times higher LOD than previous ones based either on WLRS [32] or integrated onto silicon MZIs [34]. (b) The instrumentation used is battery-operated, as both the LED and the spectrophotometer are powered by the laptop, ensuring autonomous operation in low-resources environment. This is also a major innovation compared to previous instrumentation set-ups. (c) The chip fabrication cost is reduced by 8–10 times compared to the cost of the chip with the array of 10 integrated MZIs [34]. Finally, since the set-up developed is an analytical platform, it can be employed for the on-site detection of other contaminants such as antibiotics in milk or harmful substances in general in food matrices.

## 3. Conclusions

The application of an immersible photonic chip sensor for the determination of AFM1 in whole milk from different animal species (cow, sheep, and goat) has been demonstrated. The fact that the sensor developed does not require fluidics and fluid circulation equipment for the performance of the assay simplifies the assay greatly, but mostly the instrumentation required. Thus, the only external components required are optical ones, i.e., a light source`+-, a spectrophotometer, and a bifurcated fiber, leading to a set-up that is really compact and lightweight, making it suitable for point-of-need applications. The developed immersible photonic chip immunosensor is a highly effective tool for detecting aflatoxin M1 (AFM1) in milk, since it achieves a high detection sensitivity involving a compact and robust system. The sensor developed is capable of detecting AFM1 at concentrations as low as 20 pg/mL, with a dynamic detection range from 50 to 2000 pg/mL and a total analysis time of 20 min. In addition, its ability to directly measure in undiluted milk from different species enhances its suitability for the on-site detection of AFM1 at farms and milk collection points. The wide acceptance of the proposed sensor by the dairy industry would be facilitated by the fact that the cost of the chip is comparable to that of the LFIAs currently used for on-site AFM1 determination, with the additional advantage of quantitative results. Thus, although the chip cost is currently about EUR 15 for small-scale production in our research facility, the transfer of production to a silicon foundry could reduce the cost by at least 10-fold, i.e., at EUR 1.5. In addition, for applications such as on-site food analysis, the cost can be further downsized through the regeneration and reuse of a single bio-functionalized chip. Thus, it is expected that the developed immunosensor and measuring system could help to ensure compliance with regulatory limits for AFM1 in dairy products and could evolve into a valuable asset for improving food safety and protecting public health. To accomplish this goal, a thorough investigation of the long-term storage stability and performance of the functionalized chip under real-world testing, as well as all the measuring system tests required for approval by regulatory authorities, should be performed. Furthermore, medium-scale production to obtain a more accurate estimation of the chip’s cost is essential in order to commercially exploit the proposed sensor and measuring system.

## 4. Materials and Methods

### 4.1. Reagents

Aflatoxin M1 (AFM1), AFM1 conjugate with bovine serum albumin (AFM1–BSA), bovine serum albumin (BSA), goat polyclonal anti-rabbit IgG antibody, 3-aminopropyl-triethoxy silane (APTES), glutaraldehyde solution (25% in water), and L-tyrosine were purchased from Sigma-Aldrich (Darmstadt, Germany). Hydrofluoric acid (HF; 50% in water) was obtained from Technic Inc. (Saint-Denis, France). Streptavidin, streptavidin labeled with AlexaFluorTM 546, 3-sulfo-succinimidyl-6-[biotinamido]hexanoate (Sulfo-NHS-LC-biotin), sulfosuccinimidyl-3-(4-hydroxypheynyl) propionate (sulfo-SHPP), sulfuric acid (H_2_SO_4_), and BCA Protein Assay Kit were from Thermo Fisher Scientific (Waltham, MO, USA). The rabbit polyclonal anti-AFM1 antibody was from AntiProt (Puchheim, Germany). All other chemicals and reagents were obtained from Merck (Darmstadt, Germany). The water used throughout the study was doubly distilled. The pasteurized cow (3.5% fat), sheep (1.7% fat), and goat (3.5% fat) milk were products of Larisa Dairy S.A. “OLYMPUS” (Larisa, Greece) purchased from the local market. Fresh cow, sheep, and goat milk was received from small farms in the Attica area (Greece). Sodium azide was added to all milk samples at a final concentration of 1% *w/v*, and the samples were then aliquoted and kept at −20 °C until use. All milks were tested with AgraQuant^®^ Aflatoxin M1 High Sensitivity ELISA kit (Romer Labs GmbH; Butzbach, Germany) to confirm the absence of detectable amounts of AFM1. The goat anti-rabbit IgG antibody was biotinylated following a previously published protocol [31].

### 4.2. Instrumentation

**Figure 9:**
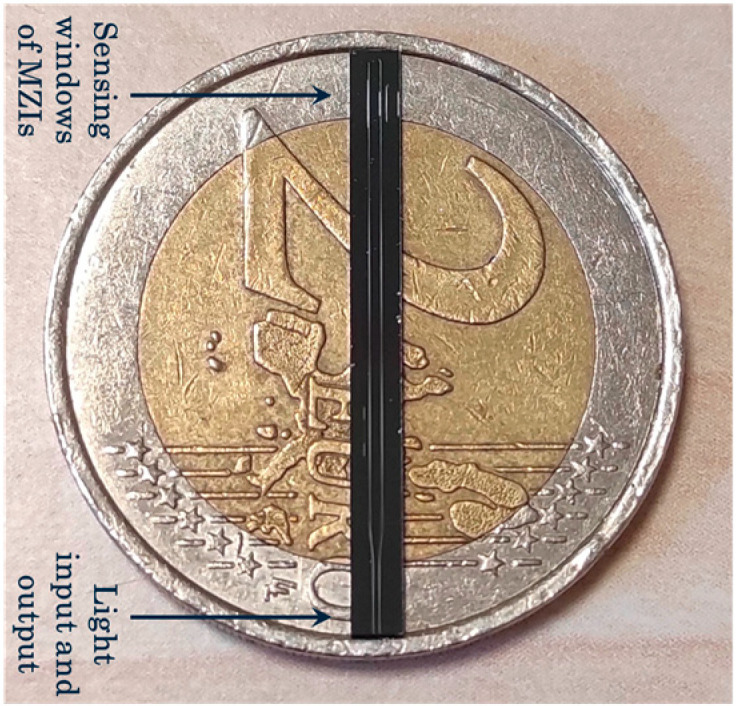
Image of the immersible photonic chip on top of a EUR 2 coin.

The photonic sensor chips were fabricated as previously described [41]. In summary, the fabrication process included the following: (a) the deposition of a silicon nitride (Si_3_N_4_) layer 150 nm thick on silicon wafers with a 5 μm thick SiO_2_ layer (under cladding layer), (b) e-beam lithography and dry etching to define the two U-shaped waveguides and the respective MZIs, (c) the deposition of a 2 μm thick SiO_2_ top cladding layer, and (d) the opening of the sensing windows through optical lithography and wet etching. The two sensing windows had a width of 20 μm and lengths of 1 mm and 2 mm, while the final chips had dimensions of 23 mm × 2.1 mm (Figure 9).

The optical system comprised a high-brightness, broad-band white light source (Ushio Europe B.V., Oude Meer, The Netherlands) and an external VIS-NIR spectrophotometer (Flame-T-VIS-NIR, Ocean Insight, Duiven, The Netherlands). This set-up connected to the chip via a bifurcated optical fiber with a 200 μm core (Ocean Insight, Duiven, The Netherlands) through the specially designed coupler. A laptop with an in-house developed software was utilized for recording signals and processing data (Figure 3a). During the assay, the dual MZI transmission spectrum was continuously captured and processed using Fast Fourier Transform (FFT) to reveal the wavenumbers of the spectra so as to track the phase shift of each MZI in real time.

### 4.3. Chemical Functionalization of the Sensor

Two different chemical activation protocols and BSA–AFM1 immobilization procedures were examined. The first one involved the modification of chips with 3-aminopropyl-triethoxy silane (APTES) and the physical adsorption of the BSA–AFM1 conjugate, and the second treatment involved HF solution and glutaraldehyde followed by the covalent bonding of the BSA–AFM1 conjugate.

#### 4.3.1. Modification with APTES

The chips were treated in an ultrasonic bath for 10 min sequentially with acetone and isopropanol, followed by Piranha treatment (1:1 volume mixture of H_2_SO_4_ and 30% *v/v* H_2_O_2_) for 20 min, washing 3 times with distilled H_2_O, and drying with a N_2_ steam to clean their surfaces. Then, the chips were immersed in a 2% (*v/v*) APTES solution in absolute ethanol solution for 60 min, washed with ethanol, dried with nitrogen, and then heated at 120 °C for 20 min. The chips were kept for at least 48 h in a desiccator prior to biological functionalization.

#### 4.3.2. Treatment with HF and Glutaraldehyde

The chemical functionalization of the chips with HF and glutaraldehyde was performed following a protocol from the literature [37], which led to the selective introduction of amine groups onto the silicon nitride layer at the window areas of the two MZIs with respect to the surrounding silicon dioxide cladding layer. In brief, after cleaning the chips with acetone and isopropanol and conducting the Piranha treatment, as described in Section 4.3.1, the side of the chip with the sensing windows was immersed for 3 min in a 1% *v/v* aqueous HF solution, followed by extensive washing with H_2_O. After that, the chips were treated for 2 h with a 2.5% *v/v* glutaraldehyde solution in 10 mM phosphate-buffered saline (PBS), pH 7.4, washed 3 times with PBS solution, and dried with N_2_. The modified chips were used immediately for spotting.

### 4.4. Determination of the Amine Content of the Chemically Functionalized Surfaces

The determination of the amine content of Si_3_N_4_ surfaces that had been modified either with APTES or with HF was performed following the modification of a previously published protocol [38]. In brief, a 20 mM sulfo-SHPP solution was prepared in 0.1 M sodium bicarbonate buffer, pH 8.5. One milliliter of this solution was added into tubes containing Si_3_N_4_ chips with dimensions of 5 mm × 15 mm and incubated under gentle shaking for 1 h at RT. Chips with APTES or HF treatment were assayed, whereas chips without any treatment were used as a blank. The chips were washed three times with 2 mL of distilled water and twice with 2 mL of 0.1 M sodium carbonate buffer, pH 11.25. The BCA working solution was then prepared following the instructions of the manufacturer by mixing 50 volumes of Component A with 1 volume of Component B, and 1mL was added to tubes containing the chips. The tubes were then incubated for 60 min at 60 °C under gentle shaking. After that, 100 μL of solution was received from each tube and transferred into a 96-well microtiter plate to determine the optical density at 560 nm using the Victor3 1420 Multilabel Counter (PerkinElmer, Waltham, MO, USA). L-Tyrosine solutions with concentration ranging from 0.1 to 2 mM were also prepared in 0.1 M sodium bicarbonate buffer, pH 8.5, and used to obtain the calibration curve. For this purpose, 50 μL from each L-tyrosine solution was mixed with 1 mL of BCA working solution and incubated for 60 min at 60 °C. Then, 100 μL of each solution was transferred into a 96-well microtiter plate and the optical density at 560 nm was measured. For the calibration curve, the optical density value of the L-tyrosine calibrators was plotted versus the L-tyrosine concentration in the calibrators (4.76–95.2 nmol/tube), and the concentration of the amine groups on the chips’ surfaces was determined first as an L-tyrosine concentration in mM and then as the actual number of amine groups per surface area using the following formula:

$$Amine\;groups\;per\;{cm}^{2}=\frac{L-tyrosine\;concentration\;in\;nmol/tube}{0.75\;{cm}^{2}}\times 6.023\times 10^{23}$$

### 4.5. Biological Functionalization of the Sensor

The 2 mm long sensing arm window of the one MZI of the chemically functionalized chips was modified with a 50 μg/mL AFM1–BSA conjugate solution in 50 mM carbonate buffer, pH 9.2, to be used as a working sensor, while the 1 mm long sensing window at the second MZI was modified with a 50 μg/mL BSA solution in the same buffer to serve as the reference sensor. The two solutions were deposited onto the respective MZIs using a microarray spotter (BioOdyssey Calligrapher Mini Arrayer; Bio-Rad Laboratories Inc.; Hercules, CA, USA) with a solid pin of a 375 μm diameter (Arrayit Corp.; Sunnyvale, CA, USA). After spotting, the chips were incubated overnight under controlled humidity conditions (65–70%), washed, and kept dry until use.

### 4.6. Assay for Detection of AFM1 with the Sensor

The chip coupled to the bifurcated fiber was immersed in a sequence of solutions placed in microtiter wells (350 μL per well). After equilibration in 50 mM Tris-HCl buffer, pH 7.8, 0.9% *w/v* NaCl, 0.5% *w/v* BSA, 5% *v/v* methanol (assay buffer/MeOH), the chip was immersed for 9 min in a 1:1 volume mixture of calibrators prepared in milk or milk samples with a 50 ng/mL rabbit polyclonal anti-AFM1 antibody solution in 50 mM Tris-HCl buffer, pH 7.8, 0.9% *w/v* NaCl, 0.5% *w/v* BSA, 10% *v/v* methanol (antibody dilution buffer), followed by a 2 min washing step in a 50 mM Tris-HCl buffer, pH 7.8, 0.9% *w/v* NaCl, 5% *v/v* methanol, and 1 min of washing with 50 mM Tris-HCl buffer, pH 7.8, 0.9% *w/v* NaCl, 0.5% *w/v* BSA (assay buffer). Then, the chip was immersed for 5 min in a 10 μg/mL biotinylated secondary antibody solution in assay buffer, washed for 1 min in the same buffer, and then immersed for 2 min in a 10 μg/mL streptavidin solution in assay buffer. After the end of the assay, the chip was regenerated by immersion for 2 min in a 50 mM HCl solution, and then for 2 min in a 0.5% *w/v* SDS solution in 0.1 M HCl, pH 1.3. Finally, the chip was washed with 50 mM Tris-HCl buffer, pH 7.8, 0.9% *w/v* NaCl, and equilibrated in assay buffer/MeOH, before the next assay cycle. A schematic of the competitive AFM1 immunoassay steps is presented in Figure 3b.

For each calibrator, the net signal was determined by the difference of the signal obtained from the working sensor minus that of the reference sensor multiplied by 2, as the length of the reference sensor window was half (1 mm) compared to the window of the working sensor (2 mm). The signal used to prepare the AFM1 calibration curve was that received during the 2 min reaction with streptavidin (analytical signal). The calibration curve was plotted as the percent ratio of each calibrator signal (S_x_) to the zero calibrator signal (S_0_) versus the AFM1 concentration in the calibrators.

## Figures and Tables

**Table 1:** Recovery of known amounts of AFM1 spiked in full-fat cow, sheep, and goat milk.

Sample	Amount Added (ng/mL)	Amount Determined * (ng/mL)	%Recovery **
Cow milk	0.15	0.16 ± 0.01	107 ± 6.7
	0.40	0.36 ± 0.02	90.0 ± 5.0
	1.5	1.4 ± 0.05	93.3 ± 3.3
Sheep milk	0.15	0.14 ± 0.01	93.3 ± 6.7
	0.40	0.42 ± 0.01	105 ± 2.5
	1.5	1.6 ± 0.07	107 ± 4.7
Goat milk	0.15	0.13 ± 0.01	86.7 ± 6.7
	0.40	0.45 ± 0.03	112 ± 7.5
	1.5	1.4 ± 0.10	93.3 ± 6.7

* Mean value ± S.D. ** Percent mean value ± S.D.

**Table 2:** Comparison of the MZI immunosensor developed with other label-free biosensors for determination of AFM1 in dairy products.

Detection Principle	Biorecognition Element/Assay Type	Sample	LOD (pg/mL)	Assay Duration (min)	Ref.
**Electrochemical sensors**					
Fe_3_O_4_/polyaniline film on IDE	Aptamer/direct binding	Buffer	1.98	60	[14]
Dendrimer layer on a gold platform	Aptamer/direct binding	Milk (pretreatment required)	8.47	60	[15]
Neutravidin layer on a gold platform	Biotinylated aptamer/direct binding		8.52		
Poly(neutral red) and carboxylated pillar[5]arene on GCE	Aptamer/direct binding	Cow/Sheep milk Kefir (pretreatment required)	40	60	[16]
MoS_2_/UiO-66-modified screen-printed carbon electrodes	Antibody/direct binding	Milk (pretreatment required)	60	10	[20]
Silver wire electrode	Antibody/direct binding	Milk (pretreatment required)	1	20	[21]
**Optical sensors**					
LSPR	Aptamer/direct binding	Milk (pretreatment required)	40	60	[27]
LSPR based on TIRE	Aptamer/competitive with Au nanoparticles labeled secondary antibody	Buffer	10	15	[30]
Surface Plasmon Resonance (SPR)	BSA–AFM1 conjugate/competitive immunoassay	Milk/ Milk powder (pretreatment required)	100	10	[31]
White Light Reflectance Spectroscopy	BSA–AFM1 conjugate/competitive with biotinylated secondary antibody and streptavidin	Milk	6	25	[32]
Fab’ functionalized asymmetric MZIs	Antibody/direct binding	Milk (pretreatment required)	16.8	1.5	[33]
Broad-band MZIs	BSA–AFM1 conjugate/competitive with biotinylated secondary antibody and streptavidin	Milk Chocolate milk (pretreatment required) Yogurt (pretreatment required)	5 5 10	15	[34]
Immersible photonic chip	BSA–AFM1 conjugate/competitive with biotinylated secondary antibody and streptavidin	Milk	20	20	This work

## Data Availability

The original contributions presented in this study are included in the article/supplementary materials. Further inquiries can be directed to the corresponding author.

## Supplementary Materials

The following supporting information can be downloaded at: https://www.mdpi.com/article/10.3390/toxins17040165/s1, Figure S1: (a) Real-time signal response for the zero AFM1 calibrator in assay buffer. The sequence of solutions is as follows: start to arrow 1: assay buffer; arrow 1 to 2: anti-AFM1 anti-body solution/zero calibrator mixture in assay buffer; arrow 2 to 3: assay buffer; arrow 3 to 4: biotinylated secondary antibody in assay buffer; arrow 4 to 5: assay buffer; arrow 5 to end: streptavidin in assay buffer. (b) Real-time signal response for the zero AFM1 calibrator in undiluted cow milk. The sequence of solutions is as follows: start to arrow 1: assay buffer; arrow 1 to 2: anti-AFM1 antibody solution/zero calibrator mixture; arrow 2 to 3: washing buffer; arrow 3 to 4: assay buffer; arrow 4 to 5: biotinylated secondary antibody in assay buffer; arrow 5 to 6: assay buffer; arrow 6 to end: streptavidin in assay buffer. In both cases the black line corresponds to the reference MZI response, the red to the working MZI, and the blue line is the difference of the two responses (net chip signal); Figure S2: Fluorescence microscope image depicting the area of the windows over the two MZIs from a chip functionalized with HF/glutaraldehyde after running the AFM1 assay and using, at the final assay step, Alexa FluorTM 546 labelled streptavidin. The image was acquired with an Axioskop 2 Plus epifluorescence microscope (Carl Zeiss; Hamburg, Germany) facilitated with an appropriate filter pair and a MicroPublisher 3.3 RTV CCD camera (QImaging, Surrey, BC, Canada) for image acquisition, and processed with the Image ProPlus software (Media Cybernetics, Inc.; Rockville, MD, USA); Figure S3: L-tyrosine calibration curve obtained with the BCA protein assay method. Each point is the mean value of three replicates ± SD; Figure S4: Net zero calibrator signals obtained from chips spotted with different concentrations of AFM1-BSA conjugate when 50 ng/mL concentration of anti-AFM1 was employed for 9 min. Each column corresponds to the mean of 3 chips ± SD; Figure S5: Typical calibration curve of AFM1 in ewe (blue line) and in goat milk (orange line). Each point is the mean value of 3 measurements ± SD; Figure S6: Calibration curves of AFM1 (black line), AFB1 (purple line), AFB2 (orange line), and AFG1 (blue line) obtained with the immersible MZI immunosensor coated with AFM1-BSA conjugate. The dashed vertical lines correspond to analyte concentration that provides 50% inhibition (horizontal dashed black line); Figure S7: Net zero calibrator signals obtained from chips stored at RT and assayed over a period of 8 weeks. Each column corresponds to the mean of 3 chips ± SD. Horizontal solid lines correspond to mean value ± 3SD.
