# Supplementary material for: Aflatoxin M1 Determination in Whole Milk with Immersible Silicon Photonic Immunosensor
Source: Toxins (Basel). 2025 Mar 26;17(4):165. doi: 10.3390/toxins17040165 (PMC12031367; doi:10.3390/toxins17040165)
Supplement: Supplementary file 1 [file toxins-17-00165-s001.zip › toxins-3518043-supplementary.pdf]

toxins

Supplementary material

Aflatoxin M1 Determination in Whole Milk with Immersible Silicon Photonic Immunosensor

Dimitra Kourti, Michailia Angelopoulou, Eleni Makarona, Anastasios Economou, Panagiota Petrou, Konstantinos Misiakos and Sotirios Kakabakos

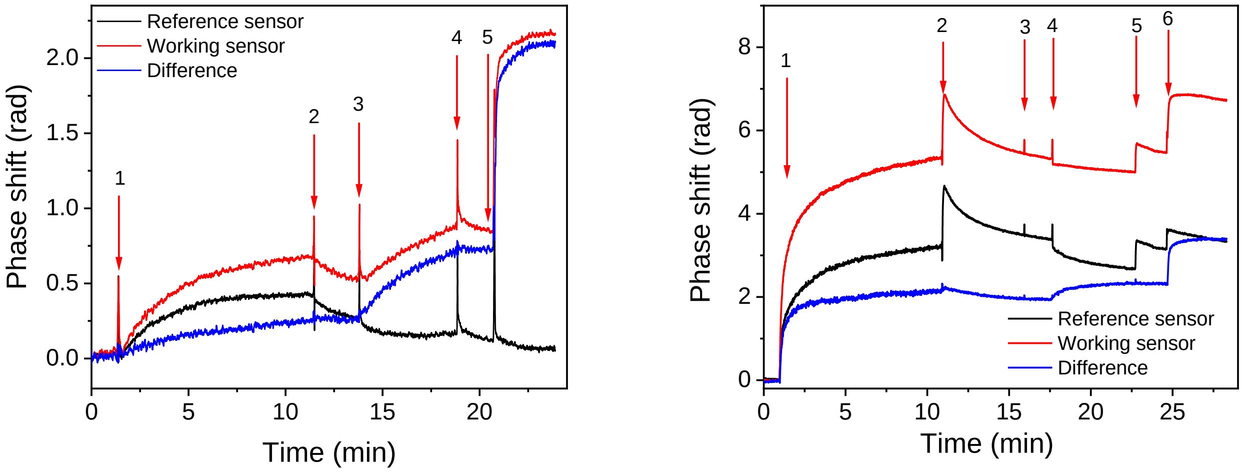

(a) **(b)**

Figure S1. (a) Real-time signal response for the zero AFM1 calibrator in assay buffer. The sequence of solutions is as follows: start to arrow 1: assay buffer; arrow 1 to 2: anti-AFM1 antibody solution/zero calibrator mixture in assay buffer; arrow 2 to 3: assay buffer; arrow 3 to 4: biotinylated secondary antibody in assay buffer; arrow 4 to 5: assay buffer; arrow 5 to end: streptavidin in assay buffer. (b) Real-time signal response for the zero AFM1 calibrator in undiluted cow milk. The sequence of solutions is as follows: start to arrow 1: assay buffer; arrow 1 to 2: anti-AFM1 antibody solution/zero calibrator mixture; arrow 2 to 3: washing buffer; arrow 3 to 4: assay buffer; arrow 4 to 5: biotinylated secondary antibody in assay buffer; arrow 5 to 6: assay buffer; arrow 6 to end: streptavidin in assay buffer. In both cases the black line corresponds to the reference MZI response, the red to the working MZI, and the blue line is the difference of the two responses (net chip signal).

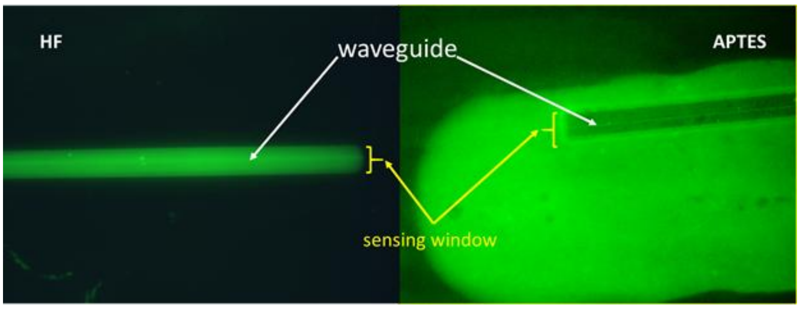

(a)

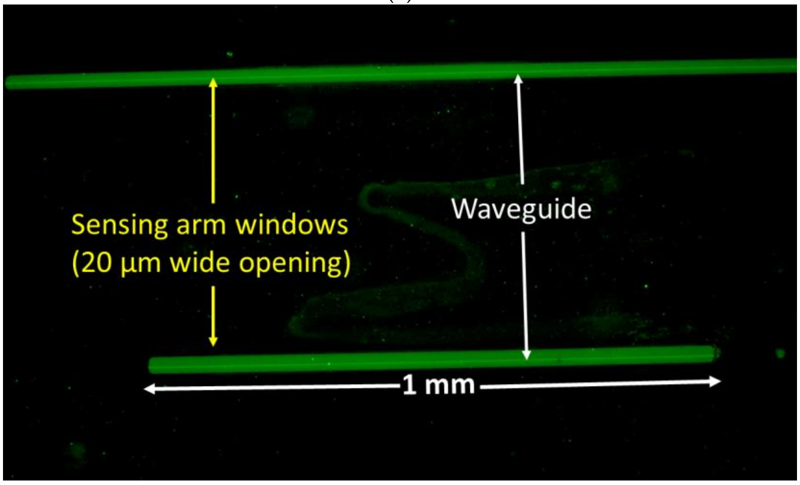

(b)

Figure S2. (a) Fluorescence microscope images depicting the area of the window over the working MZIs from chips functionalized with HF/glutaraldehyde (left) or APTES (right) after running the AFM1 assay and using Alexa FluorTM 546 labelled streptavidin. In the case of APTES modified chip, the fluorescence expands beyond the sensing window in the whole surface spotted with BSA-AFM1, while in the HF/glutaraldehyde modified chip, fluorescence is restricted to sensor window. (b) Fluorescence microscope images depicting the area of the windows over the two MZIs from a chip functionalized with HF/glutaraldehyde after running the AFM1 assay and using Alexa FluorTM 546 labelled streptavidin.

The images were acquired with an Axioskop 2 Plus epifluorescence microscope (Carl Zeiss; Hamburg, Germany) facilitated with an appropriate filter pair and a MicroPublisher 3.3 RTV CCD camera (QImaging, Surrey, BC, Canada) for image acquisition, and processed with the Image ProPlus software (Media Cybernetics, Inc.; Rockville, MD, USA).

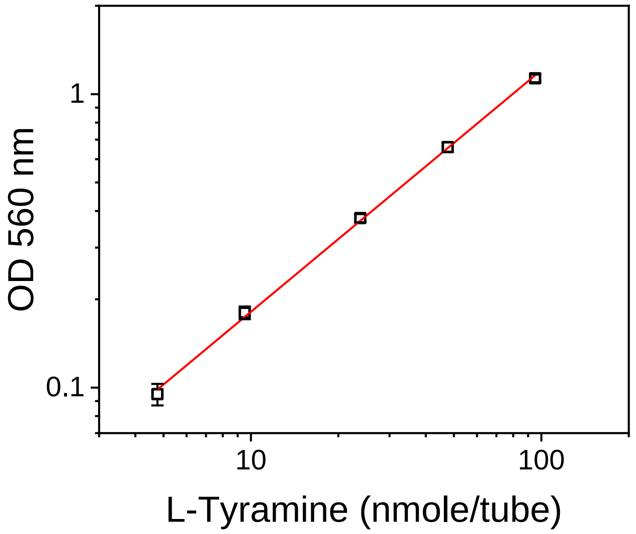

Figure S3. L-tyrosine calibration curve obtained with the BCA protein assay method. Each point is the mean value of three replicates ± SD.

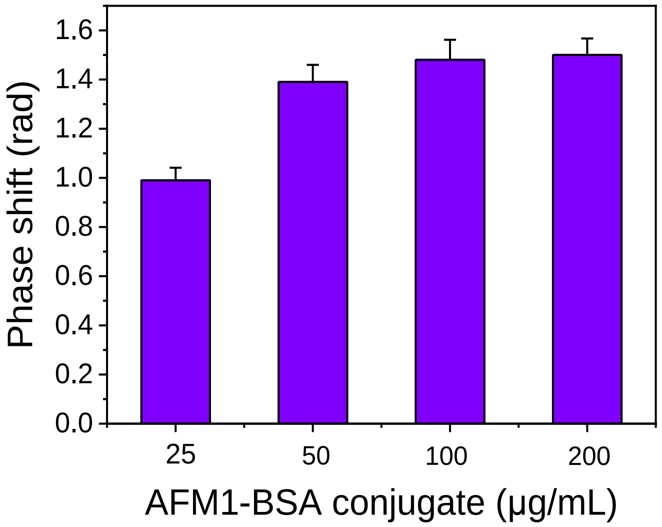

Figure S4. Net zero calibrator signals obtained from chips spotted with different concentrations of AFM1-BSA conjugate when 50 ng/mL concentration of anti-AFM1 was employed for 9 min. Each column corresponds to the mean of 3 chips ± SD.

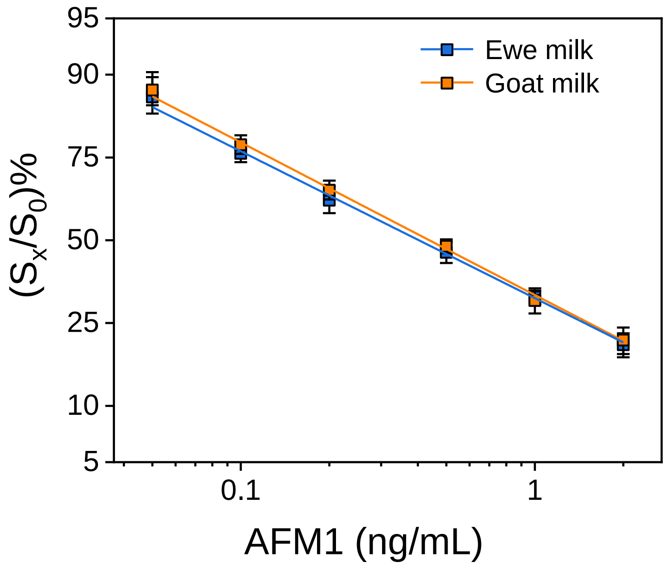

Figure S5. Typical calibration curve of AFM1 in ewe (blue line) and in goat milk (orange line). Each point is the mean value of 3 measurements ± SD.

Cross-reactivity determination

The assay specificity was assessed through cross-reactivity experiments involving other aflatoxins with similar chemical structure with AFM1 such as aflatoxin B1 (AFB1), aflatoxin B2 (AFB2), and aflatoxin G1 (AFG1). Therefore, stock solutions of the above aflatoxins were used for the preparation of calibrators with concentration ranging from 0.1 to 1000 ng/mL. The percent cross-reactivity (%CR) was calculated from the calibration curves obtained with the tested cross-reactants with respect to the calibration curve of AFM1 according to the equation:

$$\%CR = \left[\frac{IC_{50}\,AFM}{IC_{50}\;cross-reactant}\right] * 100$$

where IC_{50} AFM1 is the concentration of AFM1 providing 50% inhibition, and IC_{50} cross-reactant is the concentration of the tested aflatoxin corresponding to 50% inhibition of the respective zero calibrator signal. The results are depicted in Figure S6. The cross-reactivity values determined were 1.4% for AFB1 and 0.30% and 0.12% for AFB2 and AFG1, respectively.

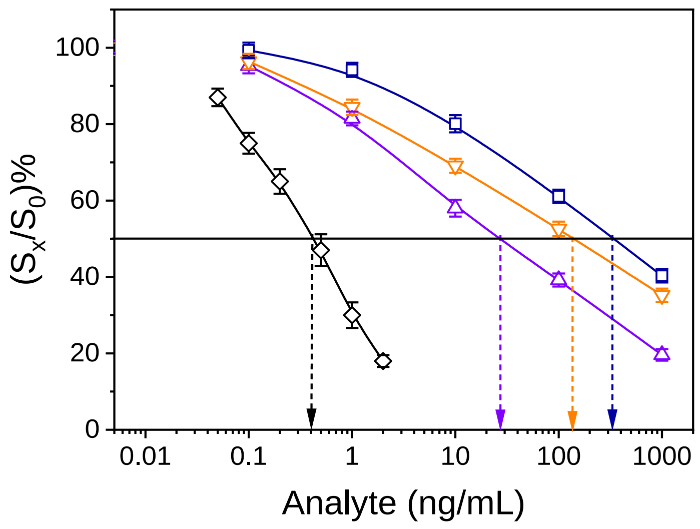

Figure S6. Calibration curves of AFM1 (black line), AFB1 (purple line), AFB2 (orange line), and AFG1 (blue line) obtained with the immersible MZI immunosensor coated with AFM1-BSA conjugate. The dashed vertical lines correspond to analyte concentration that provides 50% inhibition (horizontal dashed black line).

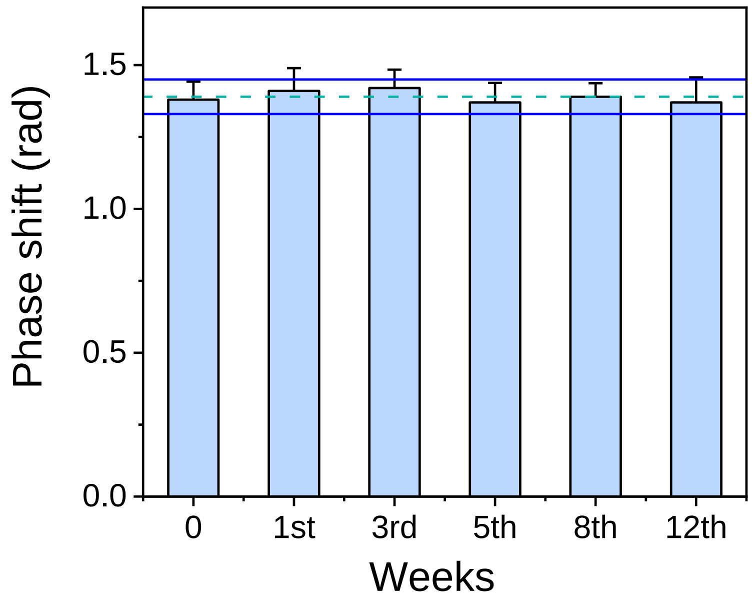

Figure S7. Net zero calibrator signals obtained from chips stored at RT and assayed over a period of 12 weeks. Each column corresponds to the mean of 3 chips ± SD. Horizontal solid lines correspond to mean value ± 3SD.
